# Evaluation of Methodologies for Assessing Self-Healing Performance of Concrete with Mineral Expansive Agents: An Interlaboratory Study

**DOI:** 10.3390/ma14082024

**Published:** 2021-04-17

**Authors:** Chrysoula Litina, Girts Bumanis, Giovanni Anglani, Marta Dudek, Riccardo Maddalena, Maria Amenta, Stamatoula Papaioannou, Gloria Pérez, José Luis García Calvo, Eloy Asensio, Rubén Beltrán Cobos, Fabiano Tavares Pinto, Algirdas Augonis, Robert Davies, Ana Guerrero, Mercedes Sánchez Moreno, Teresa Stryszewska, Ioannis Karatasios, Jean-Marc Tulliani, Paola Antonaci, Diana Bajare, Abir Al-Tabbaa

**Affiliations:** 1 Department of Engineering, University of Cambridge, Trumpington Road, Cambridge CB2 1PZ, UK; aa22@cam.ac.uk; 2 Institute of Materials and Structure, Faculty of Civil Engineering, Riga Technical University, LV-1658 Riga, Latvia; girts.bumanis@rtu.lv (G.B.); diana.bajare@rtu.lv (D.B.); 3 Politecnico di Torino, Department of Structural, Geotechnical and Building Engineering, Corso Duca degli Abruzzi, 24, 10129 Turin, Italy; giovanni.anglani@polito.it (G.A.); paola.antonaci@polito.it (P.A.); 4 Chair of Building Materials Engineering, Faculty of Civil Engineering, Cracow University of Technology, 31-155 Cracow, Poland; marta.dudek@pk.edu.pl (M.D.); tstryszewska@pk.edu.pl (T.S.); 5 School of Engineering, Cardiff University, Cardiff CF24 3AA, UK; maddalenar@cardiff.ac.uk (R.M.); daviesre11@cardiff.ac.uk (R.D.); 6 Institute of Nanoscience and Nanotechnology, National Centre for Scientific Research “Demokritos” (NCSRD), 15341 Agia Paraskevi, Greece; m.amenta@inn.demokritos.gr (M.A.); s.papaioannou@inn.demokritos.gr (S.P.); i.karatasios@inn.demokritos.gr (I.K.); 7 Institute for Construction Sciences Eduardo Torroja (IETCC-CSIC), 28033 Madrid, Spain; gperezaq@ietcc.csic.es (G.P.); jolgac@ietcc.csic.es (J.L.G.C.); eloyadl@ietcc.csic.es (E.A.); aguerrero@ietcc.csic.es (A.G.); 8 Department of Inorganic Chemistry and Chemical Engineering, University of Córdoba, Campus de Rabanales, 14071 Córdoba, Spain; rbeltran@uco.es (R.B.C.); ftavares@uco.es (F.T.P.); msmoreno@uco.es (M.S.M.); 9 Faculty of Civil Engineering and Architecture, Kaunas University of Technology, Studentu St. 48, LT-51367 Kaunas, Lithuania; algirdas.augonis@ktu.lt; 10 Politecnico di Torino, Department of Applied Science and Technology, Corso Duca degli Abruzzi, 24, 101029 Turin, Italy; jeanmarc.tulliani@polito.it

**Keywords:** round robin, self-healing concrete, standardization, expansive minerals, crack sealing, durability

## Abstract

Self-healing concrete has the potential to optimise traditional design approaches; however, commercial uptake requires the ability to harmonize against standardized frameworks. Within EU SARCOS COST Action, different interlaboratory tests were executed on different self-healing techniques. This paper reports on the evaluation of the effectiveness of proposed experimental methodologies suited for self-healing concrete with expansive mineral additions. Concrete prisms and discs with MgO-based healing agents were produced and precracked. Water absorption and water flow tests were executed over a healing period spanning 6 months to assess the sealing efficiency, and the crack width reduction with time was monitored. High variability was reported for both reference (REF) and healing-addition (ADD) series affecting the reproducibility of cracking. However, within each lab, the crack width creation was repeatable. ADD reported larger crack widths. The latter influenced the observed healing making direct comparisons across labs prone to errors. Water absorption tests highlighted were susceptible to application errors. Concurrently, the potential of water flow tests as a facile method for assessment of healing performance was shown across all labs. Overall, the importance of repeatability and reproducibility of testing methods is highlighted in providing a sound basis for incorporation of self-healing concepts in practical applications.

## 1. Introduction

Cracking in concrete is a common sight, resulting from mechanical loading or deformation-induced stresses during its service life. Although these cracks may not directly compromise the integrity of the structure, they can significantly accelerate its degradation. Cracks can create direct paths for ingress of aggressive agents into the concrete, resulting in corrosion of the reinforcing steel and thus limiting the service life. To ensure the designed service life and remediate the defects of the structure, repair actions need to be undertaken. However, those repair regimes tend to be costly, time-consuming, impractical, and often untimely due to the remote location of the defects in the structure. It has been estimated that half of the annual EU construction budget is allocated to the repair of existing structures [1], whilst an exponential growth of demand of concrete repairs exists [2].

Worldwide increasing awareness for sustainable use of natural resources and reduction of CO_2_ emissions has made evermore apparent the need to ensure the service life and performance of concrete infrastructure as a means to reduce the impact of the construction industry [3]. In this context, self-healing technologies able to repair or even prevent defects could reduce the influence of cracking on the degradation of concrete infrastructures, extending their service life. The ability of concrete and cement-based materials to intrinsically self-seal cracks is long established [4,5]. Systematic studies and emerging research activity in the last two decades has allowed the development and validation of a range of techniques to promote and enhance self-healing capacity of cement-based materials [6]. Although the concepts and mechanisms of autogenous and autonomic healing have been defined and acknowledged [7,8], from a design and application perspective, it is necessary to evaluate the effectiveness of different self-healing technologies based on their intended application [9]. However, up until now, a standard framework for comparison amongst different studies was lacking [10]. This is further hindered by the numerous experimental variables that can affect the reported self-healing behaviour [9].

To pave the way towards incorporation of self-healing concepts to design practices and address the need for standardization of testing methods for assessing the effectiveness of different technologies, six different interlaboratory tests have been undertaken under the framework of the EU COST Action 15,202 SARCOS [11]. A secondary focus of these collaborative efforts is to also assess and quantify the performance of different proposed healing technologies in concrete. Previous work reported in literature has focused in paste or mortar [6,12,13], inadvertently neglecting to account for effects of dilution of healing agents on the self-healing efficiency as their addition is typically bound to cement fraction. To better reflect on the range of healing mechanisms developed in the literature and allow for a more comprehensive assessment, within the remit of the SARCOS Action, each of the six round robin tests focused on a different self-healing technique: (1) concrete with mineral additions, (2) concrete with the addition of magnesium oxide, (3) concrete enhanced with crystalline admixtures, (4) high-performance fibre-reinforced concrete enhanced with crystalline admixtures, (5) concrete with preplaced macrocapsules containing polymeric healing agent [10], and (6) concrete with encapsulated bacteria.

The interlaboratory test reported here is one of three within the framework of the COST Action SARCOS that consider the use of mineral additions on the self-healing performance of concrete. Minerals with expansive actions have been found to not only promote self-sealing but also the recovery of mechanical properties (self-healing), e.g., [14,15,16,17,18,19]. This paper reports on the interlaboratory tests on concrete with the addition of an expansive mineral blend based on magnesium oxide. A blend of three different powder minerals was used to enhance the healing performance: magnesium oxide (MgO), hydrated lime (L), and bentonite (B). Magnesium oxide was selected as the main healing agent due to its expansion potential and compatibility with the cementitious matrix [20] and has been found to encourage the formation of brucite and other magnesium hydro-carbonate products [21]. Different studies have already shown good results for the same levels of MgO addition in the mix [17,18,21,22,23,24,25,26,27,28]. This was combined with hydrated lime and bentonite. The former was used as an additional source of calcium to support formation of portlandite, calcite, and calcium-based hydration products [29], whilst the latter was added as a complementary expansive mineral due to its swelling and expansive properties [14]. For this combination of minerals, the bending strength can be significantly regained (up to 67%) for early-age cracking and partially recovered (up to 45%) for 28 days initial cracking compared to 15% and 5% reported, respectively, for the control concrete [18,27]. The regain in liquid tightness (permeability) is significant—reaching up to 75% as assessed by gas permeability tests [18,27] and almost 90% according to sorptivity coefficient measurements [22]. Overall reported crack sealing of almost ~90% after 28 days of healing has been shown for cracks ranging within 0.18 ± 0.04 mm [27]. The use of the same type of MgO within the same range of content has been shown to improve crack area healing by 74–99% between 14 and 56 days of healing [22].

In total, nine labs from seven different European countries participated in this inter-laboratory test. This work aims to assess the effectiveness of experimental methodologies used for the evaluation of self-healing with mineral agents. Moreover, it provides new perspectives on the efficiency of MgO-based expansive minerals as a self-healing admixture for concrete where water-tightness is a key factor. The methodology used is based on water permeability tests, water capillary absorption tests, and crack width measurements, comparing their performance to evaluate self-healing. These tests have been predominantly used to assess autogenous and mineral-based self-healing in literature [8,9,12,30] and have been adopted consistently across all three round robin tests focusing on mineral additives. This interlaboratory test was split up in two parts: statistical investigation of the repeatability and reproducibility of the testing methods and assessment of healing performance. Thus, concrete prisms with and without mineral additions were cracked in a three-point bending test with a passive crack-width control and studied in a capillary water absorption test. Concurrently, discs with and without mineral additions were cracked using a splitting test-setup able to produce tensile cracks and subsequently exposed to water permeability test to evaluate the water flow going through the cracks. Fibres were used in the mix as internal reinforcement to control the opening [31]. The employed water permeability test is a variation of the EN 12390-8:2019 standard test that has been investigated previously to assess the sealing efficiency of concrete with expansive mineral agents [15,16]. A final complementary test was also done to assess the durability of the cracked and self-healed specimens through chloride ingress tests. After a predefined period of ponding with chloride solution, samples were sawn, and the penetration depth of chlorides was measured qualitatively via a colorimetric test by using silver nitrate.

## 2. Materials and Methods

This section provides information on the used healing agent, specimen preparation, and the executed tests. All specimens were produced in one laboratory (Lab 1) to negate the influence of local materials and production errors. An equal number of samples was sent to all laboratories with cracking and subsequent testing taking place at the participating laboratories.

### 2.1. Healing Agent

Compatible supplementary minerals can improve the self-healing capacity of traditional cement and concrete materials through increasing the formation of healing products [29]. Three types of expansive minerals, magnesium oxide (MgO), bentonite, and hydrated lime, were used in this interlaboratory study to produce a composite mix of healing additives to be added, supplementing part of the cement. The MgO (RBH Ltd., Manchester, UK) was a moderate reactive (light-burned) grade magnesia calcined from magnesite. This type of MgO contains 93.18% of MgO and was neutralized at 2.4 min in an accelerated acidic reactivity test [32]. The bentonite supplied by MKM Ltd. (Ankara, Turkey) is a montmorillonite clay containing mostly ~54.2% SiO_2_ and 18.8% Al_2_O_3_ in altering layers. The final mineral, hydrated/slaked lime (supplied by LHOIST, Bukowa, Poland), was provided as a dry white powder. The chemical composition and physical properties are presented in Table 1. The mineral blend was prepared as a ternary mix of 5% MgO, 5% slaked lime, and 2.5% bentonite (M5L5B2.5) by weight of cement with a total cement substitution of 12.5% by weight. This combination has been shown to previously deliver optimum healing performance in terms of crack width reduction and recovery of durability indicators [18,27]. Specimens containing the mineral blend were denoted as ADD specimens, as opposed to reference specimens without mineral additions which were denoted as REF specimens. The reference specimens which remained uncracked were denoted as UNCR.

### 2.2. Specimen Preparation

**Figure 1:**
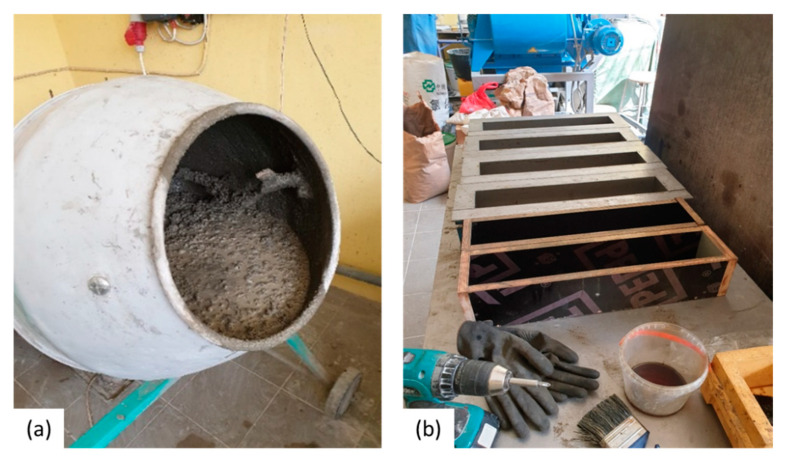
Sample preparation of concrete specimens with mineral additions ((**a**) mixing; (**b**) moulds used for casting).

Concrete prisms with a dimension of 100 × 100 × 500 mm^3^ and cylinders with a Ø100 × H200 mm were cast using the mix composition given in Table 2. The cement used was a CEM I 42.5 N (SCHWENK, Riga, Latvia), and the water-to-cement ratio was equal to 0.5 for the REF and 0.55 for the ADD. The maximum aggregate size was 16 mm. Mix was designed for a slump consistency of S2 as defined in BS EN 12350-2 with superplasticizer content adopted accordingly. Steel fibres (Dramix 65/35 BN from Bekaert, Zwevegem, Belgium with a length of 35 mm and an aspect ratio of 55) were added in all mixes as reinforcement to control cracking. The dry components were first mixed for 3 min, after which 70% of the water was added and mixed for a further 3 min (Figure 1). Then, 25% of the water together with the superplasticizer were introduced, mixing continued for another 3 min, and finally the rest of the water was added to the mixture and mixed for 2 min. For each lab, a separate batch was made to cast all specimens. All specimens were compacted with a hand-held concrete vibrator. The specimens were stored in a curing room (20 °C and >95% RH), and the day after casting they were demoulded. The specimens were sealed in plastic foil in groups of 3 to prepare them for shipping.

For the different batches, the fresh density (BS EN 12350-6:2019) was determined. Additionally, from the same batch as the test specimens also control cubes with a side of 100 mm were cast to determine the concrete compressive strength (EN 12390-3:2019). The cubes were demoulded at the same time as the test specimens and kept in water until testing at 28 days. Before shipping, the ultrasonic pulse velocity (UPV) (EN 12504-4:2004) test was conducted on both prisms and cylinders to assess the quality of the samples and complement compressive strength results. The strength and UPV testing for all specimens happened at Lab 1. All participating labs received the same number of REF and ADD specimens.

### 2.3. Experimental Methodology

#### 2.3.1. Damage Initiation: Precracking Process

Prior to cracking the prism specimens, the different participating labs stored them in water up to the age of 28 days from casting and then sawed a notch with a depth of 10 ± 2 mm in the bottom of the specimens at the middle of the span. At an age of 1 month after casting, the specimens were cracked in a three-point bending test with a span of 300 mm. Such a schedule was followed by five labs out of the total of nine. However, due to unexpected delays with deliveries and laboratory access, Lab 3, 7, and 8 started testing at 2 months after casting and Lab 9 at 6 months. As a consequence, three different ages of initial cracking could be considered, and therefore the influence of aging on the reactivity and availability of the mineral healing agents could be also explored.

**Figure 2:**
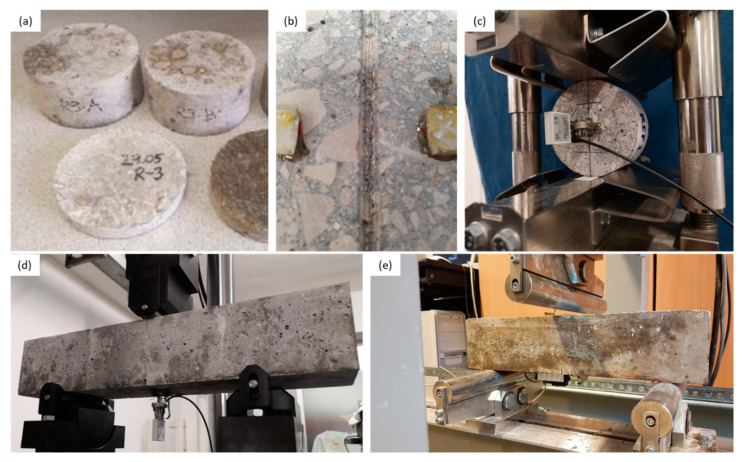
Sample preparation of specimen for testing ((**a**) discs extracted from cylinders; (**b**) notches created on either side of discs; (**c**) example of testing setup for cracking for crack control) with different closed-loop feedback systems for cracking of prism specimen; (**d**) CMOD control; and (**e**) LVDT control).

Depending on the lab, the crack formation was controlled using a closed-loop feedback system by means of either a linear variable differential transformer (LVDT) or a crack mouth opening displacement (CMOD) clip gauge mounted on the bottom of the specimens or by digital image correlation (DIC) measurements. The crack was opened at a speed of approximately 0.7 μm/s. The intended crack width at the crack mouth at loaded state was 300 μm. The samples are then unloaded manually or by a programmed rate. In an unloaded state the target crack width (at the crack mouth) was around 200 μm. Table 3 gives details on the feedback system used. Three reference specimens were kept uncracked (UNCR) for control testing. From all cylindrical concrete specimens, the different participating labs cut three discs of Ø100 × H50 mm, discarding the ends of the cylinder (Figure 2). Two notches, symmetrically on either side, (~5 mm depth) were introduced in the middle of discs. All discs were precracked by splitting test (at a loading speed of 0.7 μm/s) reaching a crack width of 200 ± 50 μm after unloading (around 300 μm in loaded state). The testing procedure was adapted from [27,33], where residual cracks of 200 ± 30 μm were considered.

**Figure 3:**
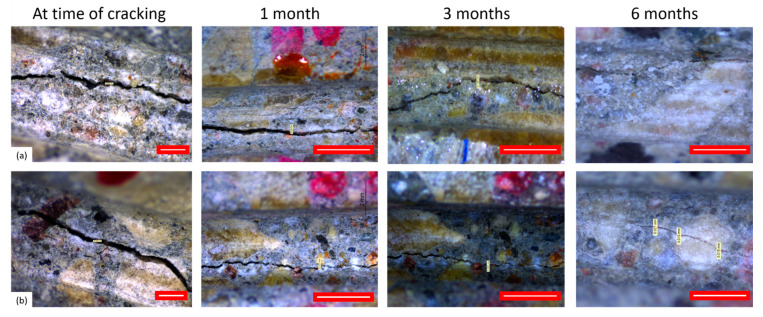
Indicative images of crack healing at time of cracking and at different healing periods for (**a**) REF and (**b**) ADD specimen. A gradual reduction of the crack width can be seen with increase of healing period. However, no precipitation of healing products was observed. (Scale 1 mm).

After initial precracking and between tests, all the samples were stored submerged in water to promote the self-healing reactions during three predefined periods of 1, 3, and 6 months. The same samples were tested before healing, and at the abovementioned three monitoring periods to monitor the healing process. Indicative images of healed samples are given in Figure 3. The self-healing specimens with mineral additions were kept in separate water containers with respect to the reference samples without additions. All prisms were stored with the crack facing downwards to avoid further crack opening. The disks were stored vertically. Crack widths were observed through optical microscopy, and the crack mouth healing was calculated thereafter. Along the crack path, different locations (six for disc specimens and four for prism) were chosen to measure the crack width. In each location, the crack width was measured five times. The reported average crack width was calculated as the average of the dataset compiled from all measuring points over the different locations of a crack. Crack-mouth healing (CMH) was then calculated as follows:

(1) $$CMH=\frac{CW_{unhealed}\left(ti\right)-CW_{healed}\left(t\right)}{CW_{unhealed}\left(ti\right)}\times 100\%$$

where *CW_unhealed_* is the crack width of the unhealed specimens at time *t_i_* (i.e., immediately after precracking) and *CW_healed_* is the crack width of the specimen after time *t_t_* (i.e., after the generic healing period). An overview of the testing program adopted to evaluate self-healing, with details of the testing sequence and healing intervals is given in Table 4.

#### 2.3.2. Capillary Water Absorption

Prior to capillary water absorption, prism specimens were dried in an oven at 40 °C for a minimum of 14 days until constant weight was achieved. Constant weight was achieved when the change in mass over a period of 2 h was less than 0.2%. The specimens were subsequently stored for 1 day at approximately 20 °C and 60% RH. Prior to testing, the specimens were partially waterproofed using aluminium tape. The bottom of the specimens was completely waterproofed except for a zone on the bottom with a width of 14 × 100 mm^2^ centred on the crack. The sides of the specimens were also sealed up to a height of 30 mm including the sides of the specimen where the crack is located to prevent the influence of small waves when specimens were removed or placed back in the water. For each prism, specimen the dry weight was recorded, and subsequently the prisms were placed in containers partially filled with water. The specimens were placed on spacers so that water could circulate under the sample. The water level in the containers was approximately 5 ± 1 mm above the notch. During a period of 24 h (at time 0 and after minutes: 1, 16, 36, 49, 64, 81, 100, 121, 144, 169, 196, 225, 256, 289, 324, and 1444), the mass of each of the prisms was measured. Care was taken so that the excess water on the surface was removed before testing by a slightly prewetted cloth.

The results were plotted in a graph (*x*-axis: √time (√h), *y*-axis: water infiltration (mm)). The slope of the line is termed the sorption coefficient SC. Self-sealing ability is then evaluated with this method on a minimum of three specimens per series (ADD and REF) as follows:

(2) $$SE=\frac{SC_{unhealed}\left(t_{i}\right)-SC_{healed}\left(t\right)}{SC_{unhealed}\left(t_{i}\right)}\times 100\%$$

where, *SE* the sealing efficiency, *SC_unhealed_* the sorption coefficient for unhealed specimens at time (*t_i_*) (initial time), and *SC_healed_* the sorption coefficient after time (*t_t_*).

#### 2.3.3. Water Flow Test

**Figure 4:**
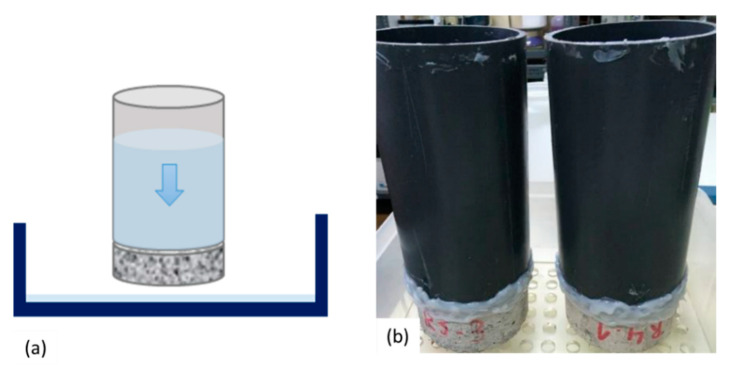
Water permeability setup measured with falling head setup; (**a**) graphical representation of setup and (**b**) experimental setup used.

To measure the water permeability of the specimens, a water flow test was proposed based on a variation on the method by [15,16]. Prior to executing the test, specimens were stored at 40 °C for at least 1 day. A PVC tube of at least 200 mm height was glued on the top of one the faces of the discs using a resin (or silicon glue) and allowed to dry for at least 24 h. The sides of the discs were sealed to avoid leakage from the side of the cracks and isolate any observed flow through the crack at the bottom of the disc (Figure 4). The tube was then filled with 1.5 L of tap water, and the timing started hereafter. The water head dropped freely in such a way that water could flow from the tube through the crack, from where it could leak out of the specimens. Only the water leaking out of the crack mouth, i.e., the bottom side of the specimens, was considered. The time needed for the 1.5 L to flow through was recorded. If the time exceeded 20 min, then the drop of water level at 30 min from the start of the test was measured.

The sealing efficiency $SE_{flow}$ of a healed specimen with respect to its unhealed (damaged) state was calculated as:

(3) $$\;SE_{flow}=\frac{V_{unhealed}\left(t_{i}\right)-V_{healed}\left(t\right)}{V_{unhealed}\left(t_{i}\right)}\times 100\%$$

where *V_unhealed(ti)_* is the volume of water that passed through the specimen’s unhealed crack at time (*t_i_*) and *V_healed(t)_* is the volume of water that passed through the specimen’s healed crack after the healing period time (*t_t_*).

#### 2.3.4. Durability of Healed Concrete against Chloride Penetration

**Figure 5:**
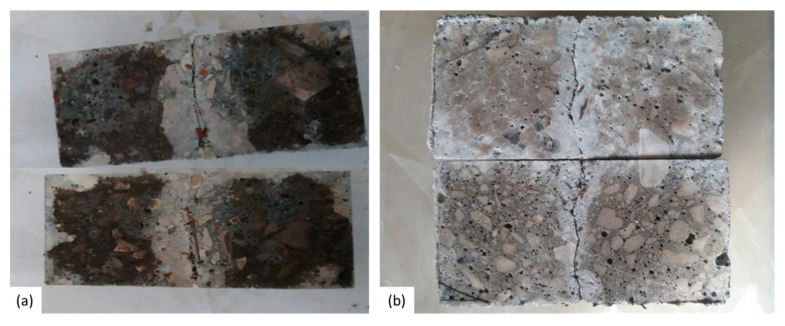
Indicative chloride penetration colorimetric assessment of (**a**) REF and (**b**) ADD specimen.

To complement water permeability measurements, chloride depth penetration was also evaluated to assess the sealing efficiency in preventing or reducing the ingress of chlorides. This test was performed only on samples that were characterized as completely healed through water permeability test. After the each of the prescribed healing periods, discs of the ADD series that showed complete healing were used. Maximum three disks were tested at each monitoring period. In this way, a minimum of three discs was allowed to continue healing until 6 months. The three best-performing REF discs were selected for chloride testing to be compared to the ADD samples. The same PVC tube setup that was used for the water permeability tests was adopted here. However, for the chloride ingress test, the tubes were filled with a chloride solution of NaCl (33 gr/L), and the samples were allowed to saturate for 3 days. Subsequently, the discs were cut perpendicular to the crack plane, and silver nitrate was sprayed on the section as an indicator. Samples were then placed in an oven at 50 °C for 1 day. Silver ions (Ag^+^) and free chloride ions (Cl^−^) react forming silver chloride (AgCl), leading to a white precipitation, whilst when reacting with hydroxyl ions, a silver oxide precipitation is formed [34] (see Figure 5 as an example).

To help quantify the penetration of chlorides through the crack, the coloration change in regions with presence of free chlorides was determined via machine learning by using the Trainable Weka Segmentation plugin in the open source software ImageJ (Fiji version 1.52) [35]. After manually training the machine learning algorithm, it was possible to produce a pixel-based segmentation of the areas where white AgCl precipitation formed. The segmented images were then filtered to remove outliers such as aggregates, after which the images were manually checked for misidentified zones. The surface penetration depth was not considered, only the ingress from the healed crack; thus, the ability for the healed section to hinder the ingress of chlorides could be evaluated. The application of the Trainable Weka Segmentation to analyse images within the context-enabling characterization of cementitious components shows great promise [10,36,37]. Once the images were manually checked, the area of chloride ingress around the crack was determined. The chloride ingress ratio was then defined as the area with chloride over the total area and was reported as the average from both crack faces of a specimen, as it was noted that the spread was similar on each crack face

## 3. Results and Discussion

### 3.1. Characterisation of Hardened Concrete

For each individual batch of concrete, the hardened density and compressive strength were determined, see Table 5. The mean concrete strength measured on cubes with a side of 100 mm at an age of 28 days was equal to 58.2 and 36 MPa for REF and ADD series, respectively. Concurrently, the early-age strength at 3 days was also determined for two batches to allow for the evaluation of early strength development at the time of shipment. It was noted that the compressive strength for the REF batch shipped to Lab 1 was significantly lower than the other ones. Results for ADD specimens showed a broader variation than the REF specimens and overall reduced strength both at 3 and 28 days. The high proportion of expansive mineral substitution, in particular bentonite, was then shown to drastically affect the compressive strength due to slow participation in pozzolanic reaction [18]. The reported ~38% reduction in strength confirms previous findings on the effect of this combination of mineral on strength development [18,27]. To further assess the variation in apparent strength at the time of shipping (7 days), UPV tests were conducted on both prisms and cylinders for the REF and ADD series for each batch (Table 6). Results for REF and ADD confirmed the compressive strength measurements with all batches revealing higher UPV values for REF compared to the ADD specimens with larger variability for the latter.

### 3.2. Cracking and Crack Width

**Figure 6:**
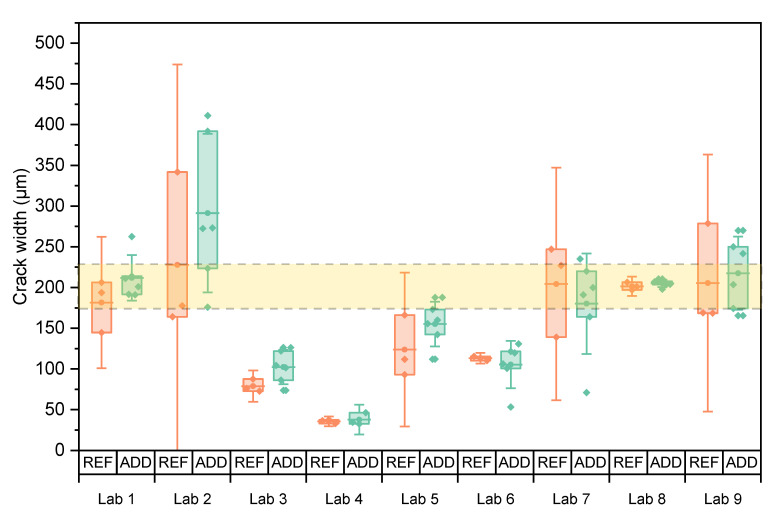
Crack width of individual prism specimens for which the mean of the series is indicated by horizontal lines and error bars give the 95% confidence interval on this mean. The area between the two dotted lines indicates the desired crack width.

Prism samples were cracked under closed-loop controlled three-point bending tests, and the residual crack widths were measured. Figure 6 shows the individual mean crack width of each specimen as well as the mean of the series and the 95% confidence interval on this mean (error bars) for both REF and ADD specimens. The area between the two dotted lines indicates the desired crack width. There is evident variation in the results. For each lab, it was statistically analysed if the mean crack width of the REF and ADD series was equal to the target crack of 200 μm (level of significance, LoS = 5%). Table 7 indicates that this hypothesis was not valid for both the REF and ADD series of Lab 3, 4, 5, and 6. In particular, Lab 4 reported the lowest crack width of all participating laboratories. Most labs used CMOD control to produce the cracks by loading to a higher crack opening (~300–350 μm) and then allowing for elastic recovery due to the presence of fibre reinforcement and closure during unloading. Lab 2 applied an LVDT controlled cracking following a similar loading pattern. When lower openings were targeted such as in the case of Labs 3–6, the load was not enough to force a larger residual crack. Moreover, lab 4 measured the crack opening on the sides of the specimens due to limitations of the microscope used for the size of the samples and the depth of the notch. This could have skewed the measurements towards smaller values. Lab 7 also reported similar difficulties, performing crack monitoring predominantly on the side of the samples rather than on the crack mouth. Considering that both labs used CMOD to control the crack mouth, it can be assumed that the overall crack under loading for Lab 4 was lower than the target 300 μm leading to a lower residual crack opening.

For each lab, independent sample t-test analysis (LoS = 5%) was conducted to assess the difference of the mean crack width of the REF and ADD series. Table 7 suggests that for all labs, results were not significantly different (*p* > 5%). Indeed Figure 6 shows that within each lab the crack width creation was repeatable, although consistently ADD series reported higher initial crack widths. This could also be a result of the reduced mechanical properties of this mixture [18,27]. Lab 6 and Lab 7 reported a reverse trend, yet the coefficient of variation (CV) of the ADD series is consistently higher than the REF specimens. Overall, a high CV was reported for both REF and ADD series in participating labs. This could be ascribed to the addition of steel fibres, since their random orientation and distribution may have affected the cracking behaviour and concurred to increase the variability in the residual crack widths.

**Figure 7:**
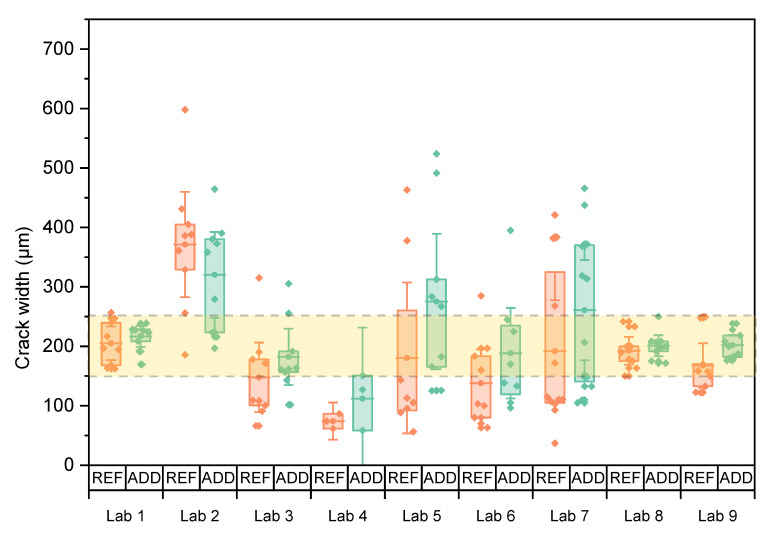
Crack width of individual disc specimens for which the mean of the series is indicated by horizontal lines, and error bars give the 95% confidence interval on this mean. The area between the two dotted lines indicates the desired crack width.

To study if there was a significant difference for the crack widths obtained by the different labs, the results of the REF and ADD specimens were taken together. The equality was investigated through ANOVA analysis of means. Equal variances were confirmed by a Levene’s test (LoS = 5%, *p* = 13.2%). Tests confirmed that means were not all equal (LoS = 5%, *p*~0%). In the post hoc analysis, a Tukey multiple comparison revealed four groups: the means of Labs 2, 9, and 8 were equal (*p*_min_ = 5.6%); the means of Labs 8, 1, 7, and 5 (*p*_min_ = 12.7%) were equal; the means of Labs 5, 6, and 3 (*p*_min_ = 30.6%); and the means of Labs 6, 3, and 4 (*p*_min_ = 26.7%) were equal. Similar results were obtained between the REF and ADD specimens (*p* = 22.4%) within each lab, and labs using a higher CMOD obtained (nearly) comparable results. The initial crack width was also assessed for the disc specimens used for the water permeability tests. Figure 7 shows individual values as well as the mean crack width of both series with the respective 95% confidence interval. The variation of crack width is higher than the one observed for the prisms even though three maximal outliers (one for Lab 5 REF, one for Lab 6 ADD, and one for Lab 2 REF) were discarded from the dataset prior to plotting this graph and subsequent statistical analysis. Because of the higher variation on the crack width, the water permeability tests were expected to be influenced.

Overall, the execution of the splitting test with passive crack width control was characterized by application difficulties. Labs reported high scattering in crack size along the crack and between the two sides of each specimen. This was reflected in the CV of the reported crack widths. For each lab, it was statistically analysed if the mean crack width was equal to the target crack width of 200 μm (LoS = 5%). Table 8 indicates that this hypothesis was not valid in the case of the REF and ADD series of Lab 2, the REF series of Lab 4, and the REF series of Lab 6. The crack width of the REF samples was equal to the ADD specimens within each lab, as verified by independent sample tests (LoS = 5%, all *p*-values > 15%). Based on this, the REF and ADD values were combined to study if there was a significant difference for the crack widths obtained between different labs. Equal variances could not be assumed (Welch’s test *p*~0%). Results showed that not all means were equal (LoS = 5%, *p* = 0%). A subsequent post hoc test (Games-Howell pairwise comparison) identified three groupings: Labs 2, 5, and 7 were equal (*p*_min_ = 5.9%); Labs 5, 7, 1, 8, 9, 3, and 6 were equal (*p*_min_ = 24.3%); and Lab 3, 6, and 4 were equal (*p*_min_ = 9.4%). Splitting tests resulted in most labs having crack widths which fell within the desired crack range with similar results obtained between ADD and REF series within each lab (LoS = 5%, *p* = 12.9%). However, it should be noted that a large variation remains in reported values, underlining the need for control of the crack width, on both sides of the specimen.

**Figure 8:**
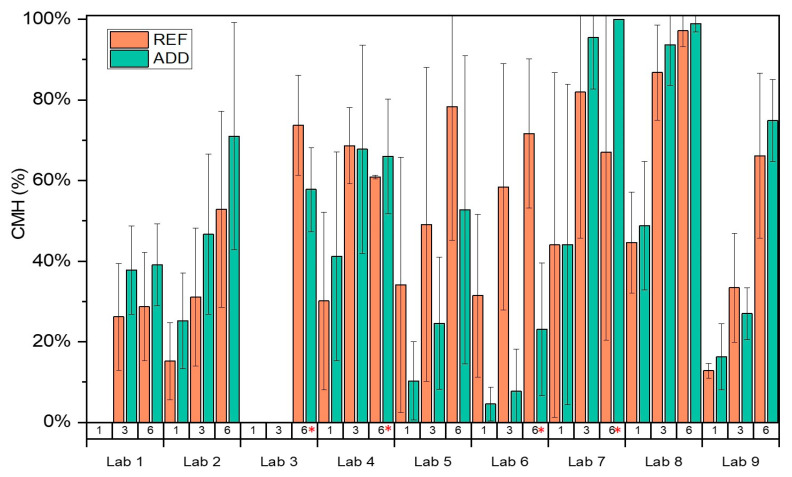
The crack healing CMH measured in the different labs. * denotes healing periods longer than the specified 6 months. Experimental work was delayed due to COVID restrictions.

Crack width was monitored for REF and ADD series by all labs over time. It should be noted here that Lab 3 measured crack widths only at *t_i_* and at the end of the final monitoring period. The crack mouth healing following Equation (1) is presented in Figure 8. Results from all concrete specimens for REF and ADD series were considered together to counterbalance the effect of the increased variability for the disc series. Overall results confirmed an improvement of observed crack sealing with time. Mean CMH increased with healing time, reaching values of 30.5%, 54%, and 66% at 1, 3, and 6 months of healing for the REF series and 27.2%, 50.1%, and 64.8% for ADD series, respectively. Statistical analysis for all the labs across all monitoring intervals confirmed no significant difference in the means of the REF and ADD series (LoS = 5%, *p* = 70.8%), with CMH for ADD series ranging from 11.8 to 80.5% and for the REF series from 27.1 to 76.2%, respectively. Further analysis of the REF series indicated that all means across labs were equal (LoS = 5%, *p* = 24.4%). However, ADD series showed higher CV (~29%) overall compared to the REF series. The higher variability reflects the higher CV in the measured crack width for the ADD series. Post hoc analysis (Tukey pairwise) identified two groups in terms of performance of the ADD series. Means CMH for Labs 8, 7, 4, 3, 2, 9, 1, and 5 were equal and above 30% (LoS = 5%, *p*_min_ = 22.1%), and, respectively, Labs 4, 3, 2, 9, 1, 5, 6 (*p*_min_ = 31.9%) were equal and between 10–50%. It should be noted that crack width of the ADD series was reportedly higher than the REF series. This could have affected the observed healing.

High levels of healing could be observed for the ADD series reaching 100% as early as 3 months for some of the participating, namely Labs 5, 7, and 8. However, the overall mean crack healing is lower than previously reported in mortar specimen [18,27] for the same content of healing agents by weight of cement for the same range of crack sizes and lower than reported by [17,21] when these minerals were introduced encapsulated in glass vials. This difference could be attributed to a dilution effect as the total content of healing agent by mass fraction is reduced as we scale up from mortar to concrete specimen. In addition, compared to previous observations [21,22], the majority of crack width reduction takes place after 1 month. Moreover, an increase in the duration of healing proved to be beneficial to the observed performance. For Lab 4 (13 months healing) and Lab 7 (10 months of healing), the presence of additions appeared to be most beneficial. Concurrently, although it could be assumed that as the matrix ages the volume of healing compounds formation decreases reducing the self-healing performance, the ADD series reported consistent healing even for older age cracking. Labs 7 and 8 reported above 70% mean crack width reduction after 6 months of healing. Similarly, Lab 9 reported CMH up to 80% for ADD series after 6 months of healing.

### 3.3. Capillary Water Absorption of Concrete

**Figure 9:**
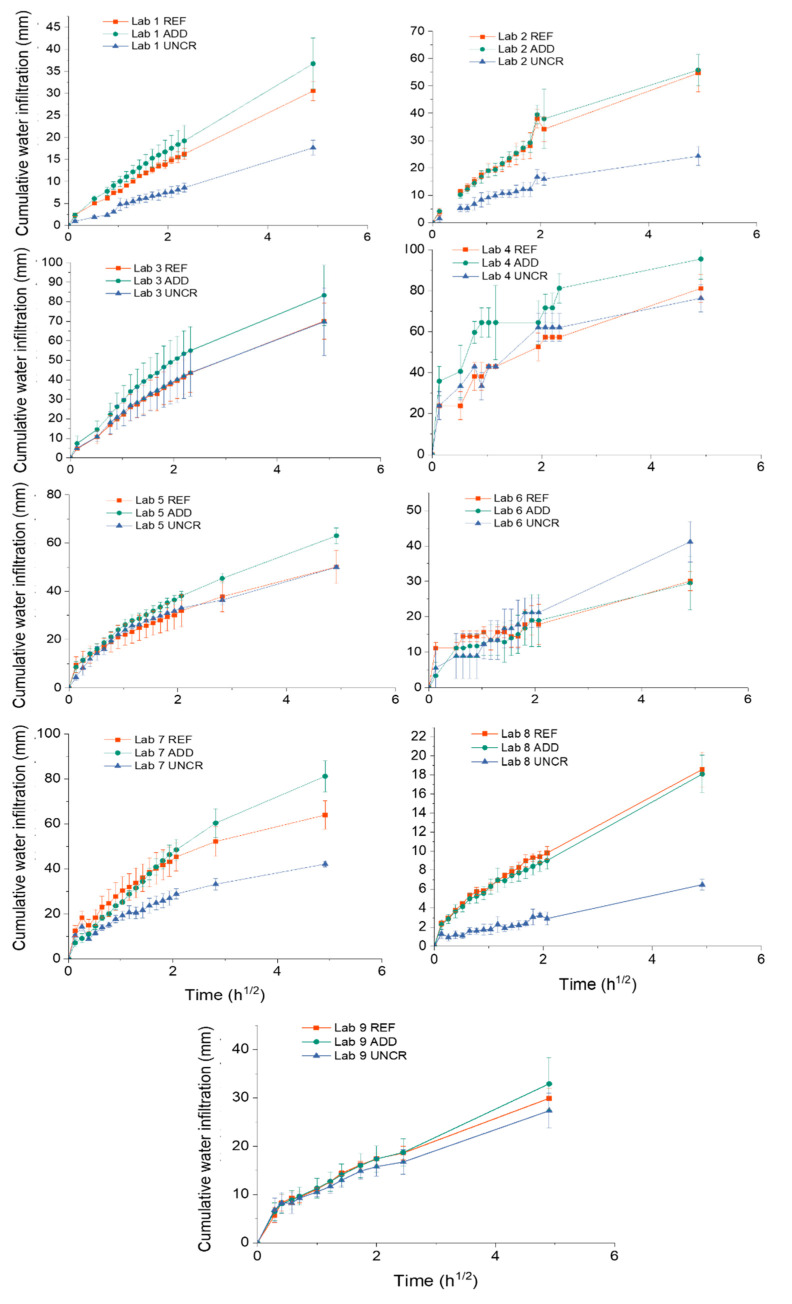
Cumulative water infiltration versus the square root of time of REF, ADD, and UNCR specimens (waterproofed with aluminium tape) of all nine labs. Error bars indicate the standard deviation.

**Figure 10:**
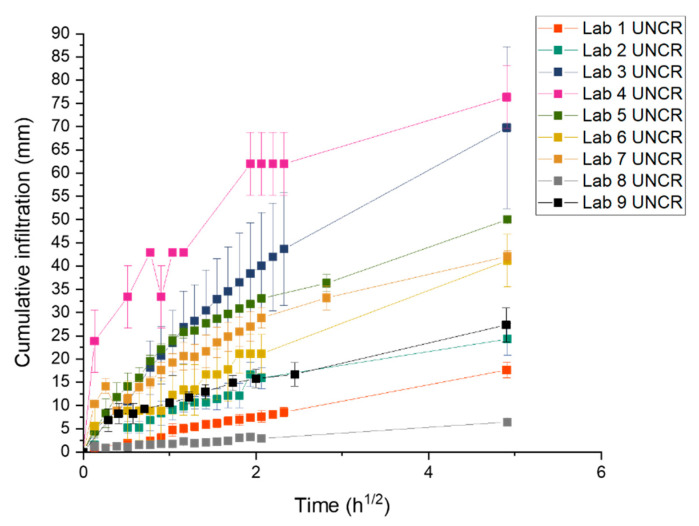
Comparison of the cumulative water infiltration versus the square root of time for the uncracked specimens of all labs showing a variation as a result of waterproofing and operating influence.

Figure 9 shows the average cumulative water infiltration for the specimens of all nine labs at time *t_i_* for REF, ADD and UNCR series. All samples were waterproofed with aluminium tape. In the case of the cracked series (REF and ADD), the average was calculated using the results of 3 and 6 specimens, respectively. For the uncracked series (UNCR), the results of 3 samples were used. Results showed significant variability between the participating laboratories. A closer look at the cumulative water infiltration for the UNCR series at *t_i_* allows for a better comparison of the repeatability and reproducibility of the method, removing the effect of the crack width opening on the behaviour of the samples (see Figure 10). Overall, most labs reported similar trends up to 6 h with exception of Lab 4 and Lab 3.

The latter exhibited almost a twofold increase of water uptake at 24 h compared to other labs for the same series. Lab 8 on the other hand showed the lowest uptake. It should be noted that the age of the specimens at the moment of initial testing (*t_i_*) was 2 months for Labs 3 and 8, and 6 months for Lab 9. All other labs performed the first capillary absorption test at the age of 1 month. A higher degree of hydration and densification of the structure could have resulted in a lower sorptivity [38]. Nonetheless, there is a significant difference in cumulative water infiltration between Lab 3 and 8 with the former reporting 12.5 times higher total infiltration than the latter. At the same time, Lab 9 reported values on the lower range of the investigated laboratories but still higher than Lab 8. The water ingress for Lab 8 might have been slightly different as these specimens were not tested with a notch. This could have reduced the overall area of the concrete in contact with the water and thus affected the observed results. However, the variation of the uncracked series of the lab, though surprising, can be explained by operator sensitivity and imperfect waterproofing. The former can be exacerbated by systematic errors and different environmental factors as previously reported by [10].

Herein difficulties were reported by most laboratories in handling the samples due to the size and weight of the specimens. The prescribed measuring intervals could be fulfilled only by adopting a time offset for the initial measurement instants, in such a way to allow for a correct handling of the specimens [10]. Moreover, the presence of sharp fibres protruding from the surface of the samples exacerbated the operating errors as it affected the quality of the waterproofing. Some labs who removed the aluminium tape immediately after testing noted that the concrete was moist in certain areas away from the crack and where the fibres had penetrated the tape. Moreover, capillary water uptake between the tape and the specimen was also frequently observed close to the sides, depending on how the tape was applied.

Comparing UNCR with REF and ADD series for all labs, it clearly appears that the presence of the crack increases the water uptake. In fact, a linear relationship between crack width and sorption coefficient has been reported [39]. Although crack widths were controlled during cracking, there is still variation on the reported ranges which will reflect on the observed water uptake. In all labs, the REF and ADD series showed higher water infiltration compared to the UNCR, with ADD series showing higher water uptake as higher crack openings have been observed. Concurrently, the presence of bentonite could account for the increase in water uptake due to its high water absorption properties [21]. However, surprisingly Lab 6 reported higher water uptake for the UNCR series compared to both REF and ADD series. This further highlights the importance of correct waterproofing and the limitations due to the use of traditional aluminium waterproofing tapes. Work completed as part of another interlaboratory testing within SARCOS (Salt Lake City, UT, USA) [10] considered the influence of the nature of waterproofing on the sorptivity results, observing a significant reduction in variability when coating with a waterproofing resin was adopted instead.

**Figure 11:**
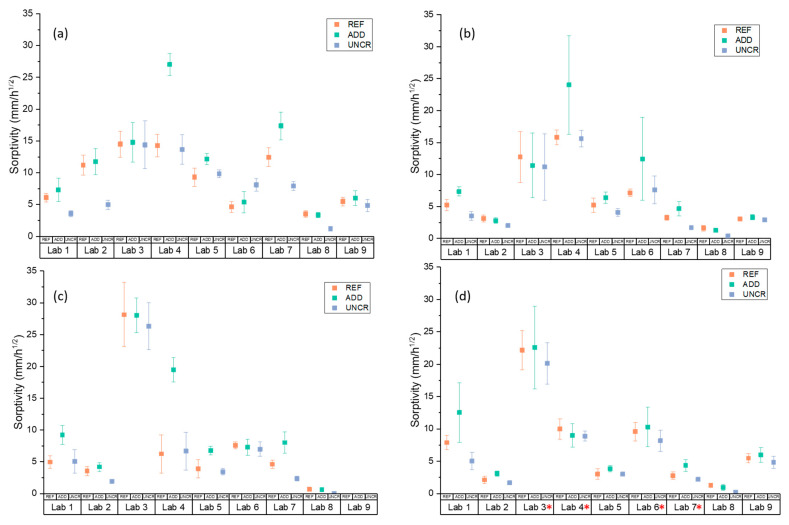
Sorption coefficients of REF, ADD, and UNCR specimens at different time intervals: (**a**) after (time 0) and after (**b**) 1 month, (**c**) 3 months, and (**d**) 6 months of healing. Error bars indicate the standard deviation. * denotes a longer healing period adopted.

**Figure 12:**
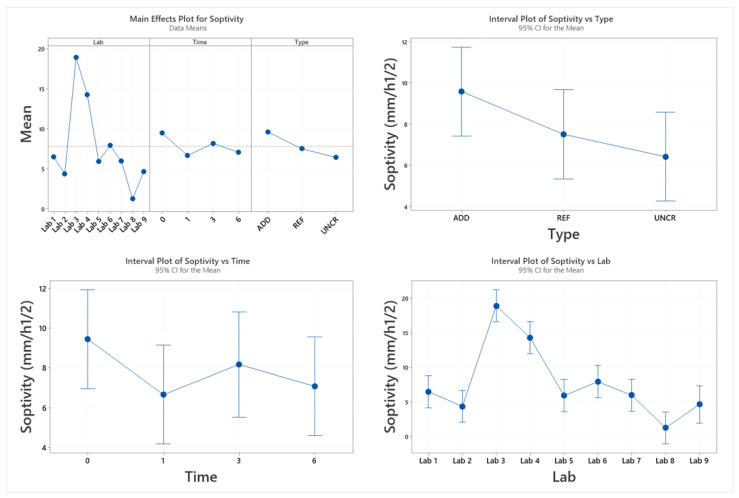
Main effects interaction plot for sorptivity coefficient.

The sorptivity coefficient was monitored as a function of healing time. It should be noted here that due to COVID-19 interruptions the last healing period was extended for some of the participating laboratories: Lab 3 (9 months), Lab 4 (13 months), Lab 6 (12 months), and Lab 7 (10 months). To calculate the sealing efficiency, the slope of linear regression curve was determined as prescribed by EN 13,057 from 10 min to 24 h. Results are reported in Figure 11. The measured sorptivity coefficient values reflected the variability observed in crack widths and testing process. For each lab, results were statistically analysed to understand the overall trends and influence of additives and healing period on the observed sorption values. Post hoc analysis (Tukey pairwise) identified three separate groups (LoS = 5%): results from Labs 3 and 4 (*p*_min_ = 12.1%); Labs 6, 1, 7, 5, 9, and 2 (*p*_min_ = 44.4%); and Labs 7, 5, 9, 2, 8, and 1 (*p*_min_ = 5.1%) were statistically equal. No lab was distinctly different. However, Labs 3 and 4 consistently reported higher sorption values compared to the other participating laboratories. Overall trends of the means (Figure 12) confirmed a general reduction of sorptivity coefficient with time. However, labs showed fluctuations of the reported sorptivity after 1 month of healing. Labs 1, 2, 3, and 7 showed the same or increased sorption coefficients between 1 and 3 months of healing. On the other hand, Labs 4, 5, and 8 showed a consistent decrease of sorption with increasing healing time for all series. Surprisingly, Lab 6 showed an increase of all observed sorption coefficients after 1 month of healing with significant variation of the results. This was attributed to an error during preliminary testing at time 0. Lab 6 was then excluded from further considerations regarding the sealing efficiency. Regardless across all labs, values after 6 months of healing confirm an improved performance for both REF and ADD series. However, when longer periods of healing are adopted (for example by Lab 3 and Lab 4), an increase in sorption coefficients is evident across all series. This was more pronounced for the ADD series. Generally, the mean sorptivity coefficient values of the ADD series were higher than the reference ones.

**Figure 13:**
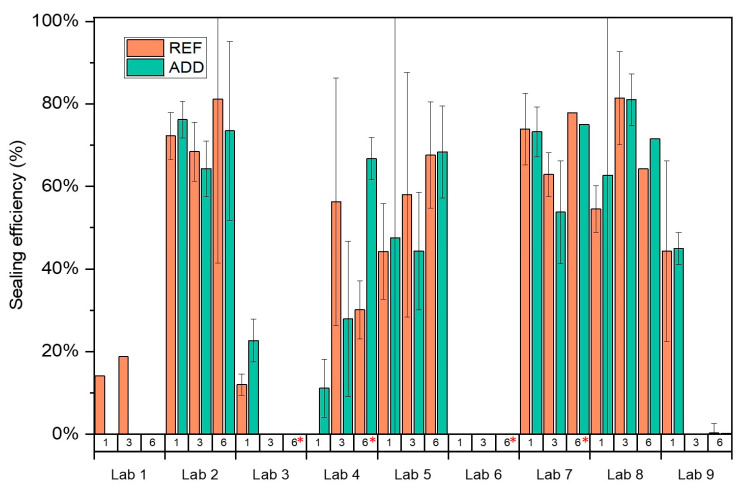
Sealing efficiency (SE) based on capillary water absorption measured in the different labs. * denotes a longer healing period adopted.

From these sorptivity coefficients, the sealing efficiency was calculated for each lab and series, as given in Figure 13. The sealing efficiency was calculated for each lab for the REF and ADD series. The results confirmed the improvement of the sealing with time in agreement with CMH observations. Statistical analysis across all labs and monitoring times, confirmed that there is no statistical difference (LoS = 5%, *p* = 93.2%) between observed sealing efficiency for the REF and ADD series. However, the presence of mineral healing additions can more consistently improve healing in the long term. In particular, after 6 months of healing, the mean SE for the ADD series ranged from 35 to 73.9%, while for the REF series, from 10 to 71.3%, respectively. This confirms previous observations by [22] on healing performance determined from sorptivity coefficient measurements for the same mineral additives. Nonetheless, compared to previous work on mortars where a higher sealing of ~90% was seen, the reported improvement herein is lower, highlighting the influence of healing agent dilution in a concrete matrix.

### 3.4. Water Permeability

**Figure 14:**
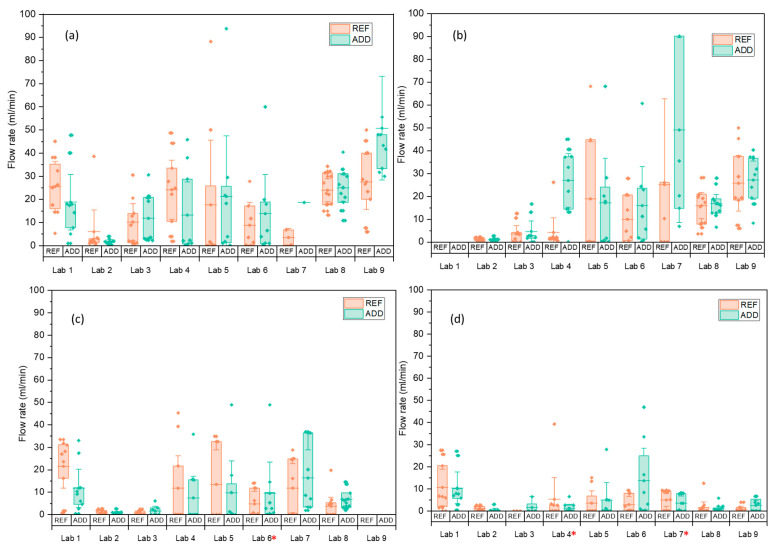
Individual flow rates q and the means of the different series indicated by horizontal lines (error bars give the 95% confidence interval on the mean); (**a**) at time 0, and after (**b**) 1 month, (**c**) 3 months, and (**d**) 6 months of healing. * denotes a longer healing period adopted.

Disc specimens (ADD and REF series) were subjected to water flow tests after cracking, at the prescribed healing time intervals. Figure 14 shows the water flow rate (mL/min) leaking from the samples during the test at time *t_i_* and after 1, 3, and 6 months of healing in water, respectively. It should be noted here that due to COVID-19 interruptions 6 months measurements were postponed for two of the participating laboratories (Lab 4 and Lab 7). Moreover, 3 months measurements could not be taken for Lab 9. The variation of the water flow was significantly higher than for the crack width; see Table 9 for comparison at *t_i_*. The crack width was measured only at the surface of the specimens, while the flow is also influenced by the internal crack geometry (tortuosity). Even for low variations of crack width, the flow variation through the crack can be a magnitude higher [40]. Moreover, labs reported difficulties controlling the crack propagation on the side of the disc specimens. Even though care was taken to waterproof and seal the sides of the crack, some water could be seen escaping from the sides giving higher flow rates, such as in the case of Lab 9. On the other hand, Lab 2 reported minimal flow rates, even though it showed the largest crack width amongst all labs. This lab observed that the acrylic sealant used to waterproof the sides had penetrated the length of the crack and sealed part of it internally. Moreover, most labs used a temperature of 40 °C to pretreat the samples for 24 h before the water permeability test. However, the pretreatment conditions could also affect the water flow influencing water absorption into the matrix. This effect of pretreatment was investigated by Lab 6. This lab reported a higher water flow at the second monitoring interval, which was attributed to the highly saturated condition of the samples between *t_i_* and 1 month. Samples at *t_i_* could be affected by storing conditions leading to higher water absorption until saturation was reached. It was then suggested that water flow measurements are done twice to allow saturation of the sample and cancel any effects of pretreatment or storage. Then, measurements were only recorded from the second run for interpretation purposes.

**Figure 15:**
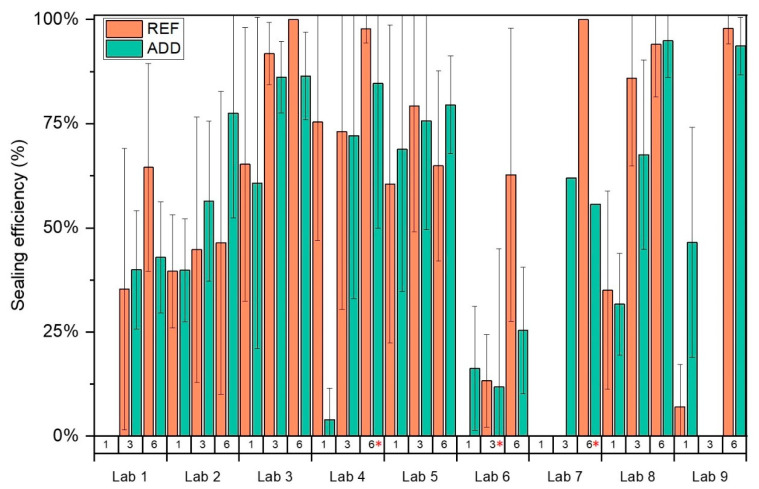
The sealing efficiency *SE_flow_* based on water flow tests measured in the different labs. * denotes a longer healing period adopted.

Considering the overall performance of both series, the sealing efficiency was calculated for all labs (Figure 15). It can be shown that in terms of SE_flow_ results of Lab 2 fall in line with the other labs. The mean sealing efficiency for the REF series varied from 25.3 to 85.7% and for the ADD series from 17.8 to 77.8%. This confirms the literature reporting up to 75% regain in liquid tightness when MgO-based minerals are considered measured, however with the gas permeability test [18,27]. There is significant improvement of healing reported with time with a more than two-fold increase from 1 to 6 months of healing. This correlates with observations for CMH and is in agreement with the previous findings by [21], reporting an accelerated rate of healing from 28 to 56 days. Moreover, the use of the same type of MgO within the same range of content has been shown previously to improve crack area healing by 74–99% between 14 and 56 days of healing [22]. Interestingly, REF and ADD series are comparable with no significant difference in the means overall (*p* = 84.3%). Nonetheless, the effect of additions appears more beneficial after 3 months of healing, with ADD series reporting consistently higher mean SE_flow_ (58.1%) compared to the REF series (51.4%). Moreover, for later age of cracking (6 months) as reported by Lab 9, the presence of additions gave significant SE_flow_ (~48%), even as early as 1 month of healing compared to the REF series (7%).

Overall, results reveal that the sealing efficiency can be promising with the ADD series with individual labs reporting even 100% healing as early as 1 month (Lab 3 and Lab 5). However, the variability needs to be controlled. The imperfect cracking had the same effect on results as reported earlier in sorptivity tests. The effect of additions on the mechanical strength leading to wider cracks hindered direct performance comparison. It is further expected that an increase in self-healing agent fraction to counteract the dilution effect could further improve the healing reported. Finally, it should be remarked that for most operators in the different labs, this was the first time to work with this kind of healing material, in this scale, and with this kind of testing method. Familiarity with technique would harmonize results.

### 3.5. Chloride Ingress

**Figure 16:**
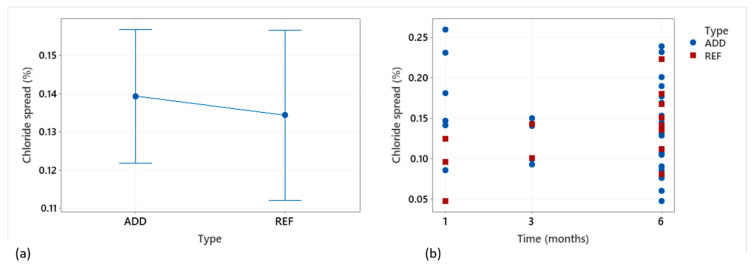
(**a**) Mean chloride surface ingress (spread) through the healed crack for REF and ADD series and (**b**) observed individual values at different time intervals. Results are based on samples that reported 100% sealing efficiency, namely complete crack sealing as assessed by water flow tests.

After performing water flow tests, Labs 1, 3, 4, 5, 7, and 8 performed chloride ingress tests on samples of REF and ADD series that exhibited 100% sealing efficiency. These tests were performed to assess the efficiency of the healed section in hindering the ingress of Cl^−^ as an indication of the potential increase of durability offered by the healing mechanism here investigated. In an effort to quantify the chloride ingress observed from images of the sawed sampled, the percentage area of chloride spread through the crack over the total area of the sample was calculated. The chloride spread is expressed numerically, but the reported values should be interpreted qualitatively. Although effort was put to standardize the procedure, subjective interpretations and errors could not be eliminated. Mean results for different healing periods from all labs are reported in Figure 16. The labs reported a similar average chloride ingress regardless of the presence of additions in the mix, 13.4% and 13.9% for the ADD and REF, respectively. Although sealing efficiency was fully recovered for all assessed samples, results indicated that the sealing in itself is not an effective indicator of the durability as it does not ensure impermeability against aggressive ions. The presence of additions did not negatively impact the performance against concrete nor was the efficiency of the healing achieved impaired compared to the REF. Nevertheless, these results underline the need to assess the durability of the healing achieved. Moreover, the impact of healing on the long-term stability and performance of the structure under a range of exposure environments needs to be considered.

## 4. Conclusions

Herein, the effectiveness of proposed experimental methodologies suited for self-healing concrete with mineral healing additions were investigated by interlaboratory testing. The study further provided information on the performance of MgO-based expansive minerals in affecting self-sealing capabilities.

Reinforced concrete specimens were cracked in a three-point bending setup controlled by closed-loop feedback system. Results revealed quite some variation in the crack width within labs. It was confirmed that labs that opened crack widths further than the recommended 300 μm were able to obtain the target crack width of 200 μm, as partial crack closure from the elastic regain due to the fibres restricted the residual crack width upon unloading. However, the random orientation and distribution of the fibres was shown to affect the cracking behaviour increasing variability. Due to large variability between the crack opening values of the same lab, it is suggested that an adequate number (e.g., 9–10) of specimens should be used, in order to reject those outside the target values. Absorption tests were executed upon cracking (and pretreatment) and after subsequent increasing healing periods spanning 6 months. Results showed a high variability between labs. This highlighted the importance of the quality of waterproofing when executing a capillary absorption test. Despite this test being extensively used in mortars and pastes to assess self-healing performance, the results can be easily affected by operator sensitivity as the sample size increases, due to difficulties with handling and managing larger samples. Even if the quality of waterproofing is improved, the size of the sample may need to be reduced for ease of handling to reduce errors.

A simple setup for water flow test was introduced. Cylindrical discs were cracked under splitting tests using CMOD to control the crack width. Most labs reported crack width that fell within the desired range (150–250 μm). Similar to the results for the controlled cracking for prisms, larger cracks widths had to be targeted to account for elastic recovery due to the presence of fibre reinforcement. However, the resulting crack widths revealed the need for control during testing on both faces of the discs. As such, the crack width of cylindrical discs was less consistent than for the prism specimen under three-point bending. This variation reflected in the water flow test results. Despite the quite large variability, none of the labs obtained a significantly different result from the others. This confirms the potential for the investigated water flow test as a suitable testing method for standardizing purposes. Further analysis of the efficiency of the achieved healing against chloride ingress highlighted that complete sealing and recovery of water tightness does not necessarily prevent the ingress of deleterious agents.

Direct comparisons between laboratories in terms of the performance of the additives is difficult and prone to error, underlining the need for appropriate testing methodologies. However, based on statistical analysis, the healing obtained by addition of MgO-based expansive agents was comparable and complementary to the reference specimens but showed greater efficiency in sustaining long-term healing and later-age crack healing as the active agents remain unreacted for longer in the matrix. The results further underlined the need to counteract the dilution effect of mineral agents for self-healing when scaling up to concrete applications. In previous studies, the efficiency of these mineral blends has been primarily demonstrated in cementitious matrices.

Despite these open issues, the knowledge developed so far demonstrates the importance of accurate damage initiation, the limitations of capillary water absorption tests to assess the sealing performance as the scale of the samples increases, the potentiality of water flow tests as a facile testing method for scaled up (in concrete) assessment of healing performance, and the need for incorporating durability testing for the assessment of any healing technology to provide a sound basis for incorporation of self-healing concepts in practical applications.

## Figures and Tables

**Table 1:** Chemical composition and physical characteristics of mineral additions.

	MgO (M)	Bentonite Clay (B)	Slaked Lime (L)
Chemical Composition			
SiO_2_ (%)	2.25	54.20	2.00
Al_2_O_3_ (%)	0.22	18.80	0.80
CaO (%)	0.87	4.90	91.12
Fe_2_O_3_ (%)	0.53	5.00	0.40
MgO (%)	93.18	3.70	0.74
SO_3_ (%)	-	-	0.1
Na_2_O (%)	-	3.00	-
K_2_O (%)	-	0.60	-
TiO_2_ (%)	-	0.70	-
CaCO_3_ (%)	-	-	-
LOI (%)	2.59	-	-
Physical Properties			
Avg. Particle size (μm)	30–40	4.75–75	-
Density (g/cm^3^)	3.02	2.80	2.24
Specific Surface area (m^2^/g)	16–20	0.48	20–25
Bulk Density (g/cm^3^)	-	-	0.4–0.5
Reactivity	145 s	-	-

**Table 2:** Concrete mix design.

kg/m^3^	Reference	Self-Healing
CEM I 42.5 N	360	315
Water	180	198
Natural Sand 0/4 mm	930	930
Crushed Dolomite Gravel 4/8 mm	530	530
Crushed Dolomite Gravel 8/16 mm	365	365
Steel Fibres	40	40
Superplasticizer	~3 L/m^3^	~3–3.2 L/m^3^
Hydrated Lime (L)	-	18
MgO (M)	-	18
Bentonite (B)	-	9

**Table 3:** Closed-loop feedback system used by the different labs and ultimate crack width during loading *W* after which specimens were unloaded.

Lab	Feedback System	MAX (μm)
1	CMOD	400
2	LVDT	350
3	CMOD	300
4	CMOD	300
5	CMOD	300
6	CMOD	300
7	CMOD	300
8	CMOD	350
9	CMOD	350

**Table 4:** Testing program to evaluate self-healing.

Self-Healing Test	Sample	Total Time of Healing *
Water Permeability	All disks	0/1/3/6 months
Chloride Penetration	Disks from water permeability (max 3 per age)	After water permeability, if no water passed through healed crack
Sorptivity	All prisms	0/1/3/6 months

* First period of healing—1 month, second period of healing—2 months (total healing of 3 months), third period of healing—3 months (total healing of 6 months).

**Table 5:** Hardened properties (hardened density and compressive strength) at 28 days of concrete batches (SD = standard deviation, NA = not available). Indicative strength at 3 days reported for two batches.

Batch Label	REF		ADD		Density, kg/m^3^	
	3 d, MPa	28 d, MPa	3 d, MPa	28 d, MPa	REF	ADD
Lab 1	NA	51.9	NA	31.3	2404	2221
Lab 2	45.4	58.8	30.9	40.7	2386	2293
Lab 3	NA	58.8	NA	32.4	2419	2233
Lab 4	NA	57.0	NA	36.3	2456	2281
Lab 5	NA	61.1	NA	38.5	2433	2330
Lab 6	NA	60.2	NA	39.4	2454	2283
Lab 7	NA	57.6	NA	36.7	2449	2303
Lab 8	NA	58.4	NA	30.6	2392	2232
Lab 9	43.1	59.5	30.6	38.3	2425	2280
Mean	44.2	58.2	30.7	36.0	2424.3	2272.8
SD	1.6	2.7	0.2	3.7	26.3	36.5

**Table 6:** Ultrasonic pulse velocity (water saturated) of concrete batches (μ = mean, SD = standard deviation, and NA = not available).

Batch Label	REF				ADD			
	Prisms, m/s		Cylinders, m/s		Prisms, m/s		Cylinders, m/s	
	μ	SD	μ	SD	μ	SD	μ	SD
Lab 1	4687	56.7	NA	NA	4143	59.9	NA	NA
Lab 2	4808	21.2	4775	8.3	4383	103.4	4437	39.6
Lab 3	4853	47.8	4735	14.8	4181	33.2	4153	55.6
Lab 4	4854	58.5	4763	26.9	4298	51.4	4330	39.2
Lab 5	5044	23.2	4736	98.2	4430	54.6	4354	48.7
Lab 6	4823	20.2	4747	42.4	4371	35.0	4411	29.7
Lab 7	4812	30.4	4771	29.4	4388	80.1	4351	13.7
Lab 8	4903	38.9	NA	NA	4252	96.0	NA	NA
Lab 9	4825	23.3	4766	23.2	4325	42.0	4312	30.3
Mean	4845		4756		4308		4335	
SD	94.5		16.5		98.5		91.8	

**Table 7:** Mean and coefficient of variation *CV* for the measured crack width of prism specimens, as well as the *p*-value for the statistical test comparing the mean to the target crack width of 200 μm, and the *p*-value for the test comparing the mean of the REF to the mean of the ADD.

Batch label	Type	Crack Width		Ref = ADD	μ = 200 μm
		MEAN	CV	*p*	*p*
Lab 1	REF	181.5	0.18	0.30254	0.56006
	ADD	212.8	0.13	-	0.47617
Lab 2	REF	227.8	0.43	0.37402	0.67495
	ADD	291.3	0.32	-	0.06084
Lab 3	REF	78.9	0.10	0.10181	0.00137
	ADD	102.3	0.20	-	7.65 × 10^−5^
Lab 4	REF	35.7	0.07	0.66671	6.98 × 10^−5^
	ADD	37.7	0.20	-	6.86 × 10^−4^
Lab 5	REF	123.7	0.31	0.18281	0.07385
	ADD	155.1	0.17	-	0.00852
Lab 6	REF	113.1	0.02	0.65673	3.10 × 10^−4^
	ADD	105.4	0.26	-	4.02 × 10^−4^
Lab 7	REF	204.3	0.28	0.57686	0.90802
	ADD	180.2	0.33	-	0.90792
Lab 8	REF	201.5	0.02	0.2916	0.94532
	ADD	205.1	0.02	-	0.97654
Lab 9	REF	205.4	0.31	0.74069	0.89611
	ADD	217.5	0.20	-	0.36283

**Table 8:** Mean and coefficient of variation *CV* for the measured crack width of disc specimen, as well as the *p*-value for the statistical test comparing the mean to the target crack width of 200 μm and the *p*-value for the test comparing the mean of the REF to the mean of the ADD.

Batch Label	Type	Crack Width		Ref = ADD	μ = 200 μm
		MEAN	CV	*p*	*p*
Lab 1	REF	205.1	0.18	0.83012	0.87845
	ADD	216.7	0.11		0.71542
Lab 2	REF	371.1	0.31	0.3191	0.00212
	ADD	320.2	0.29		0.00499
Lab 3	REF	147.8	0.52	0.30696	0.0738
	ADD	182.3	0.34		0.41382
Lab 4	REF	74.1	0.17	0.25579	0.00331
	ADD	112.1	0.43		0.08686
Lab 5	REF	233.5	0.91	0.63583	0.65036
	ADD	275.3	0.54		0.16574
Lab 6	REF	138.0	0.54	0.24757	0.03607
	ADD	188.5	0.52		0.75274
Lab 7	REF	191.9	0.70	0.21953	0.838
	ADD	260.9	0.51		0.14059
Lab 8	REF	192.4	0.16	0.51084	0.4744
	ADD	200.9	0.11		0.90632
Lab 9	REF	167.9	0.29	0.17738	0.08324
	ADD	201.7	0.11		0.82652

**Table 9:** Mean and coefficient of variation *CV* for the measured crack width *w* and water flow rate for both REF and ADD specimens of the nine labs at time 0.

Batch Label	Type	Crack Width (μm)		Flow Rate (L/min)	
		MEAN	CV	MEAN	CV
Lab 1	REF	205.1	0.18	0.02801	NA
	ADD	216.7	0.11	0.01777	NA
Lab 2	REF	371.1	0.31	0.00202	0.45
	ADD	320.2	0.29	0.00187	0.57
Lab 3	REF	147.8	0.52	0.01021	1.01
	ADD	182.3	0.34	0.01179	0.94
Lab 4	REF	74.1	0.17	0.02417	0.69
	ADD	112.1	0.43	0.01321	1.42
Lab 5	REF	233.5	0.91	0.01759	1.90
	ADD	275.3	0.54	0.02124	1.47
Lab 6	REF	138.0	0.54	0.00885	1.20
	ADD	188.5	0.52	0.01384	1.45
Lab 7	REF	191.9	0.70	0.00236	1.64
	ADD	260.9	0.51	0.00621	1.73
Lab 8	REF	192.4	0.16	0.02396	0.31
	ADD	200.9	0.11	0.02504	0.32
Lab 9	REF	167.9	0.29	0.02752	0.56
	ADD	201.7	0.11	0.05073	0.54

## Data Availability

The raw data required to reproduce these findings is available to download from: 10.5281/zenodo.4559868.
